# Shifting the balance of mitochondrial apoptosis: therapeutic perspectives

**DOI:** 10.3389/fonc.2012.00121

**Published:** 2012-10-08

**Authors:** Simone Fulda

**Affiliations:** Institute for Experimental Cancer Research in Pediatrics, Goethe-UniversityFrankfurt, Germany

**Keywords:** mitochondria, apoptosis, cancer, PI3K

## Abstract

Signaling via the intrinsic (mitochondrial) pathway of apoptosis represents one of the critical signal transduction cascades that control the regulation of cell death. This pathway is typically altered in human cancers, thereby providing a suitable target for therapeutic intervention. Members of the Bcl-2 family of proteins as well as cell survival signaling cascades such as the PI3K/Akt/mTOR pathway are involved in the regulation of mitochondria-mediated apoptosis. Therefore, further insights into the molecular mechanisms that form the basis for the control of mitochondria-mediated apoptosis will likely open new perspectives to bypass evasion of apoptosis and treatment resistance in human cancers.

## Introduction

Programmed cell death is an ancient, evolutionary highly conserved program that exists in every cell to execute cell death upon appropriate stimulation (Lockshin and Zakeri, 2007). Evasion of cell death is a hallmark of human cancers and contributes to tumorigenesis, tumor progression and treatment resistance (Fulda, 2009; Hanahan and Weinberg, 2011). Apoptosis is one of the best characterized forms of programmed cell death that plays an important role in various physiological and pathophysiological circumstances (Lockshin and Zakeri, 2007). The mitochondrial pathway of apoptosis, one of the two key apoptosis signaling pathways, is initiated by a large variety of upstream stimuli and tightly regulated by various factors including pro- and antiapoptotic proteins of the Bcl-2 family as well as the phosphatidylinositol 3′-kinase (PI3K)/Akt/mammalian target of rapamycin (mTOR) pathway (Engelman, 2009; Fulda et al., 2010). The PI3K/Akt/mTOR signaling cascade belongs to the critical survival programs that are typically overactivated in human cancers and can promote cell survival by inhibiting the mitochondrial pathway of apoptosis (Parcellier et al., 2008). The PI3K signaling network diversifies into many distinct downstream branches, one of which leads to the activation of mTOR (Shaw and Cantley, 2006). In addition, intricate interactions between distinct kinase survival networks have been described. Since small-molecule inhibitors that block PI3K/Akt/mTOR signaling are currently undergoing clinical evaluation in early trials, there is much interest to understand how these inhibitors interfere with intracellular signaling pathways, for example mitochondria-mediated apoptosis.

## Signal transduction of apoptosis

While a large variety of intracellular signaling pathways and regulatory molecules have been shown to impinge on the regulation of apoptotic programs, two major signaling cascades have emerged that represent the core machinery of apoptosis. This includes the extrinsic (cell receptor) pathway as well as the intrinsic (mitochondrial) pathway of apoptosis (Fulda and Debatin, 2006). The intrinsic (mitochondrial) apoptosis pathway is centrally integrated into a network of signal transduction cascades and is responsive to a multitude of upstream activators. Among these are cell survival pathways such PI3K/Akt/mTOR signaling, proapoptotic proteins of the Bcl-2 family, cellular stress stimuli, metabolic alterations, hypoxic conditions, or increased levels of second messenger molecules (Fulda et al., 2010). Within the mitochondrial pathway of apoptosis, the permeabilization of the outer membrane of mitochondria constitutes a key event to control downstream signal transduction pathways. Mitochondrial outer membrane permeabilization is associated with the release of mitochondrial proteins from the intermembrane space into the cytosol. This accounts for cytochrome c, second mitochondrial activator of caspases (Smac) and apoptosis-inducing factor (AIF). Cytochrome c promotes the aggregation of caspase-9 together with Apaf-1 in the cytosol to form a multi-protein complex, i.e., the apoptosome that results in caspase-9 activation. The release of Smac from the mitochondrial interspace into the cytosol promotes apoptosis by binding to IAP proteins, thereby preventing IAP protein-mediated inhibition of caspases, including caspase-3, -7, and -9 (Fulda and Vucic, 2012). AIF translocates to the nucleus to trigger large-scale DNA fragmentation in a caspase-independent manner (Hangen et al., 2010). Given the fact that permeabilization of mitochondrial outer membranes constitutes a central event with a profound impact on cellular survival, this process is tightly regulated. Pro- and antiapoptotic members of the Bcl-2 family localize to mitochondrial membranes and are involved in the control of mitochondrial outer membrane permeabilization (Adams and Cory, 2007).

## Survival signaling via PI3K/Akt/mTOR

The PI3K/Akt/mTOR pathway represents a key signal transduction pathway that mediates cell growth and blocks cell death (Shaw and Cantley, 2006). Aberrantly high activation of this survival cascade is a characteristic feature of a large variety of human malignancies and has been associated with carcinogenesis, tumor progression, treatment resistance, and poor prognosis (Engelman, 2009). Ligation of growth factor receptors by their corresponding growth factors typically results in the engagement of this survival cascade in order to activate intracellular programs that support proliferation and survival. Triggering of growth factor receptors results in the phosphorylation of these receptor tyrosine kinases that reside within the plasma membrane. This, in turn, engages activation of the whole cascade via PI3K and Akt activation. Positive output of this signaling is antagonized by the tumor suppressor gene *phosphatase and tensin homologue deleted on chromosome 10 (PTEN)*, which acts as both a lipid and a protein phosphatase (Yin and Shen, 2008). PTEN can dephosphorylate PIP_3_, thereby shutting off PI3K/Akt signal transduction.

## Akt-imposed antiapoptotic programs in cancer

**Figure 1:**
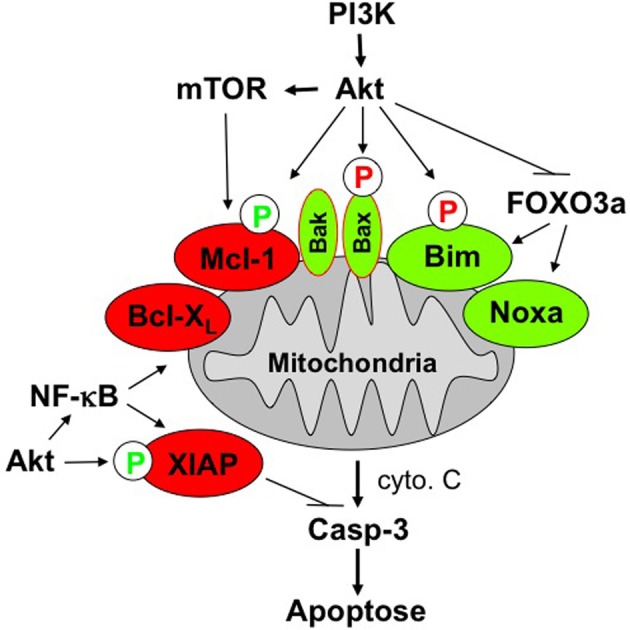
**Scheme of PI3K/Akt/mTOR-imposed antiapoptotic signaling at the mitochondria.** See text for details.

Among its various functions, Akt acts as an antiapoptotic factor that directly or indirectly antagonizes cell death signal transduction, for example via the mitochondrial pathway (Shaw and Cantley, 2006). Akt has been reported to directly interfere with cell death pathways by phosphorylating key apoptosis-regulatory proteins which, in turn, results in a shift within the ratio of pro- and antiapoptotic proteins toward the inhibition of cell death (Figure 1). One mechanism of how Akt interferes with apoptosis signaling resides in Akt-mediated phosphorylation of proapoptotic proteins which, in turn, results in inhibition of their function. This mechanism accounts for Akt-mediated inhibition of Bad, Omi/high temperature requirement protein A2 (HtrA2), caspase-9, and acinus (Parcellier et al., 2008). In addition, the multi-domain Bcl-2 protein Bax has been shown to be phosphorylated in an Akt-dependent manner at its serine residue at position 184 (Yamaguchi and Wang, 2001; Gardai et al., 2004). This Akt-mediated phosphorylation of Bax leads to a change in the conformation of Bax and blocks its activation. Besides Bax, also the Bcl-2 family protein Bim is phosphorylated at a serine residue, i.e., Ser 87 (Qi et al., 2006). Akt-stimulated phosphorylation of Bim promotes its degradation via the proteasome, thereby counteracting apoptosis induction. While the phosphorylation of the proapoptotic Bcl-2 proteins Bax and Bim reduces their proapoptotic potential, Akt-mediated phosphorylation of some antiapoptotic factors, such as XIAP and Mcl-1, vice versa decreases their antiapoptotic properties by decreasing their protein stability. Accordingly, phosphorylation of XIAP and Mcl-1 by Akt enhances the degradation of these proteins via the proteasomal machinery, resulting in a reduction of XIAP and Mcl-1 protein expression (Dan et al., 2004; Maurer et al., 2006). Apart from the direct interference of Akt with cell death signaling pathways via phosphorylation of key signal transduction molecules, Akt has also been reported to interfere with cell death programs indirectly via the phosphorylation of transcription factors. This mechanism applies to the transcription factors NF-κB and cAMP response element-binding (CREB) protein as well as forkhead transcription factors such as FOXO1, FOXO3a, FOXO4, and FOXO6. Akt-stimulated phosphorylation of these transcription factors can on one side promote the expression of antiapoptotic proteins, while on the other side it results in reduced expression levels of proapoptotic proteins. For example, Akt-mediated activation of the transcription factor NF-κB can lead to transactivation of a wide range of antiapoptotic NF-κB target genes, including Inhibitor of Apoptosis (IAP) proteins, Bcl-X_L_, and Bcl-2, just to name a few (Ozes et al., 1999; Romashkova and Makarov, 1999). By comparison, Akt-stimulated increase in the phosphorylation status of forkhead transcription factors shuts off their transcriptional activity by sequestering these transcription factors in the cytosol. This, in turn, results in reduced expression of proapoptotic proteins that are known to be regulated by forkhead transcription factors, including Bim, Noxa, tumor necrosis factor (TNF)-related apoptosis-inducing ligand (TRAIL), and FAS ligand (Van Der Heide et al., 2004). In addition, forkhead transcription factors are involved in the control of ROS levels via transcriptional regulation of antioxidant enzymes (de Keizer et al., 2011).

## Shifting the ratio of antiapoptotic toward proapoptotic factors by inhibition of PI3K/Akt/mTOR signaling

Since PI3K/Akt/mTOR signaling represents a key regulatory mechanism to control the activity of pro- and antiapoptotic Bcl-2 family proteins, small-molecule inhibitors of this signaling cascade open new perspectives to modulate the signaling outcome of the mitochondrial pathway of apoptosis. Accordingly, small-molecule inhibitors of PI3K/Akt/mTOR signaling have been reported to tilt the balance between pro- and antiapoptotic proteins toward apoptosis by decreasing the expression of Mcl-1 and by increasing phosphorylation of Bim_EL_ resulting in increased expression of Bim_EL_ (Bender et al., 2011; Opel et al., 2011). Furthermore, enhanced activation of the transcription factor FKHRL1 upon treatment with PI3K inhibitors caused increased expression levels of NOXA, as NOXA is transcriptionally activated by FKHRL1 (Obexer et al., 2007). Also, NOXA expression levels are transcriptionally controlled by Hippo/Mst1 (Valis et al., 2011). Increased expression of NOXA may promote mitochondrial apoptosis via at least two mechanisms. NOXA can directly bind to Mcl-1 thereby antagonizing the antiapoptotic function of Mcl-1 (Ploner et al., 2008). In addition, Noxa can contribute to downregulation of Mcl-1 by stimulating its degradation via the proteasome (Ploner et al., 2008).

Downregulation of Mcl-1 levels upon treatment with inhibitors of PI3K/Akt/mTOR signaling may involve posttranscriptional as well as transcriptional mechanisms. For example, activation of GSK3β upon inhibition of PI3K/Akt/mTOR signaling favors downregulation of Mcl-1 proteins, since GSK3β promotes the proteasomal degradation of Mcl-1 by phosphorylation of its serine residue at position 159 (Maurer et al., 2006). In addition, Mcl-1 is transcriptionally regulated by PI3K/Akt/mTOR signaling (Kuo et al., 2001). In addition to NOXA and Mcl-1, Bim_EL_ represents another Bcl-2 protein that is tightly regulated at the transcriptional level via PI3K/Akt/mTOR signaling. The transcription factor FKHRL1, which is turned off by aberrant PI3K/Akt/mTOR signaling, transactivates the expression of Bim_EL_, providing a molecular explanation of how inhibitors of PI3K/Akt/mTOR signaling can transcriptionally activate Bim_EL_ (Dijkers et al., 2000). In addition to this transcriptional level of regulation, Bim_EL_ is also directly controlled by Akt-mediated phosphorylation at serine residue 87 (Qi et al., 2006). This phosphorylation event stimulates the degradation of Bim_EL_ via the proteasome (Qi et al., 2006). Since Bim_EL_ can stimulate activation of Bax and Bak both directly as well as indirectly by binding to antiapoptotic Bcl-2, Bcl-X_L_, and Mcl-1, changes in Bim expression levels upon inhibition of PI3K/Akt/mTOR signaling may constitute a key event in the regulation of the mitochondrial pathway of apoptosis.

Together, a number of recent studies have provided the molecular basis for the observation that small-molecule inhibitors of PI3K/Akt/mTOR signaling represent a potent strategy to enhance the sensitivity of cancer cells toward cell death induction via the mitochondrial pathway of apoptosis. Accordingly, small-molecule inhibitors of PI3K/mTOR were shown to chemosensitize various cancers to a large variety of anticancer drugs, e.g., topoisomerase-1 or -2 inhibitors, platinum compounds, or microtubule interfering agents (Wallin et al., 2010; Bender et al., 2011; Kim et al., 2011; Mueller et al., 2012). This PI3K/mTOR-mediated chemosensitization may involve a shifted ratio between pro- and antiapoptotic Bcl-2 proteins, thereby engaging mitochondrial outer membrane permeabilization and the mitochondrial pathway of apoptosis. Overexpression of Bcl-2 antagonized this PI3K/mTOR inhibitor-stimulated activation of Bax and permeabilization of mitochondrial outer membrane and profoundly rescued cell death induction following treatment with chemotherapeutics and PI3K/mTOR inhibitors. Thus, small-molecule inhibitors of PI3K/Akt/mTOR signaling represent a promising approach to engage the mitochondrial pathway of apoptosis in order to overcome drug resistance.

## Conclusions

Mitochondrial apoptosis represents a key signal transduction pathway that is critically involved in the regulation of chemosensitivity of human cancers. Targeted modulation of mitochondrial outer membrane permeabilization, for example using small-molecule inhibitors, opens new perspectives to lower the threshold for cell death induction and to overcome at least some forms of drug resistance. This calls for further development of combination treatment strategies, aiming at lowering the threshold for mitochondria-meditated cell death induction together with additional cytotoxic strategies.

### Conflict of interest statement

The author declares that the research was conducted in the absence of any commercial or financial relationships that could be construed as a potential conflict of interest.
